# Continuous blood flow restriction during repeated‐sprint exercise increases peripheral but not systemic physiological and perceptual demands

**DOI:** 10.1002/ejsc.12106

**Published:** 2024-03-28

**Authors:** James R. Mckee, Olivier Girard, Jeremiah J. Peiffer, Alasdair R. Dempsey, Kirsten Smedley, Brendan R. Scott

**Affiliations:** ^1^ Physical Activity, Sport and Exercise (PHASE) Research Group School of Allied Health (Exercise Science) Murdoch University Perth Western Australia Australia; ^2^ Centre for Healthy Ageing Murdoch University Perth Western Australia Australia; ^3^ School of Human Sciences (Exercise and Sport Science) The University of Western Australia Perth Western Australia Australia; ^4^ Centre for Molecular Medicine and Innovative Therapeutics Murdoch University Perth Western Australia Australia; ^5^ School of Medical, Molecular and Forensic Sciences Murdoch University Perth Western Australia Australia

**Keywords:** fatigue, hypoxia, occlusion, repeated‐sprint ability, team sport

## Abstract

This study examined the impact of continuous blood flow restriction (BFR) during repeated‐sprint exercise (RSE) on acute performance, peripheral, systemic physiological, and perceptual responses. In a randomized crossover design, 26 adult male semi‐professional and amateur team‐sport players completed two RSE sessions (3 sets of 5 × 5‐s sprints with 25 s of passive recovery and 3 min of rest) with continuous BFR (45% arterial occlusion; excluding during between‐set rest periods) or without (non‐BFR). Mean and peak power output were significantly lower (*p* < 0.001) during BFR compared to non‐BFR (*d*_*z*_ = 0.85 and 0.77, respectively). Minimum tissue saturation index during the sprints and rest periods was significantly reduced (*p* < 0.001) for BFR (*d*_*z*_ = 1.26 and 1.21, respectively). Electromyography root mean square was significantly decreased (*p* < 0.01) for *biceps femoris* and *lateral gastrocnemius* muscles during BFR (*d*_*z*_ = 0.35 and 0.79, respectively), but remained unchanged for the *vastus lateralis* muscle in both conditions. Oxygen consumption and minute ventilation were significantly reduced (both *p* < 0.01) for BFR (*d*_*z*_ = 1.46 and 0.43, respectively). Perceived limb discomfort was significantly higher (*p* < 0.001) for BFR (*d*_*z*_ = 0.78). No differences (*p* > 0.05) in blood lactate concentration or rating of perceived exertion were observed between conditions. Blood flow‐restricted RSE reduced performance and likely increased the physiological and perceptual stimulus for the periphery with greater reliance on anaerobic glycolysis, despite comparable or decreased systemic demands.

## Highlights

- Continuous cuff inflation reduces external loads during repeated sprints which is suitable for athletes undertaking demanding training blocks or rehabilitating an injury.
- Blood flow‐restricted repeated sprints likely increase anaerobic energy contribution from further muscle deoxygenation.
- Inflatable cuffs likely reduce antagonistic muscle activation during cycling repeated sprints to maintain efficient inter‐muscle coordination given the deteriorated contractility of prime mover muscles with metabolic stress.

## 1 INTRODUCTION

Team sport players commonly perform repeated short‐duration sprints (≤10 s) interspersed with brief recovery periods (≤60 s) (Girard et al., 2011). Repeated‐sprint exercise (RSE) causes fatigue of peripheral (e.g., adenosine triphosphate depletion and metabolite accumulation) and neural (e.g., reduced drive to active muscle) origins (Mendez‐Villanueva et al., 2012). During RSE, team‐sport players have implemented systemic hypoxia which reduces the inspired fraction of oxygen (Beard et al., 2019), and localized hypoxia via blood flow restriction (BFR; Mckee et al., 2024). These hypoxic strategies lower oxygen availability to promote physiological adaptations thereby mitigating development of fatigue during RSE.

Localized hypoxia is induced with BFR via inflatable leg cuffs, restricting arterial blood inflow and venous return (Mckee et al., 2023). Applying BFR hinders the oxygen‐dependent resynthesis of phosphocreatine, increasing the reliance on anaerobic glycolysis during RSE (Harris et al., 1976; Mckee et al., 2023). Furthermore, oxygen consumption (V̇O_2_) is reduced during RSE performed open‐looped (continued until volitional exhaustion) and closed‐looped (fixed volume) with BFR compared to without (i.e., non‐BFR) (Mckee et al., 2024; Willis et al., 2018). Despite lower systemic V̇O_2_, investigations have reported greater heart rate (HR) during volume‐matched RSE with BFR compared to non‐BFR (Ienaga et al., 2022; Kojima et al., 2021). Thus, compensatory elevation in HR may occur during RSE with BFR to enhance cardiac output and oxygen delivery. Regardless, vascular resistance and local blood pooling caused by BFR can alter the perfusion pressure gradient, thereby limiting oxygen availability during RSE (Willis, Borrani, & Millet, 2019).

Research utilizing near‐infrared spectroscopy (NIRS) reports lower tissue saturation index (TSI) for the *vastus lateralis* muscle during RSE (3 sets of 3 × 6‐s sprints with 24 s of passive recovery and 5 min of rest) within inter‐set intermittent BFR periods only (100–120 mmHg for 2 min during rest) when compared to non‐BFR (Ienaga et al., 2022). Therefore, BFR induces localized hypoxia in active musculature during RSE. However, it is unknown if a similarly structured RSE session with continuous BFR reduces TSI during sprints and between‐sprint recovery periods. Under these circumstances, greater metabolic stress would be expected when compared to intermittent BFR, as muscle deoxygenation enhances metabolite accumulation (e.g., lactate and hydrogen ions), which can inhibit cross‐bridge function by decreasing myofibrillar calcium ion sensitivity (Mckee et al., 2023; Sahlin, 2014). However, when continuous BFR (45% arterial occlusion pressure [AOP]) is applied during open‐looped RSE (10‐s sprints with 20 s of active recovery), maximum TSI of the *vastus lateralis* muscle does not differ during sprints when compared with non‐BFR exercise (Willis, Borrani, & Millet, 2019). Similar muscle oxygenation is likely explained by substantially greater sprinting volume performed with non‐BFR compared to BFR (∼31 vs. 14 completed sprints, respectively), thereby requiring support from investigations employing volume‐matched RSE protocols.

Peripheral disturbances caused by BFR may reduce muscle activation during RSE to minimize excessive fatigue development (Girard et al., 2011). For instance, decreased (−7.8%) mean electromyography (EMG) amplitude was observed for *vastus lateralis* and *medialis* muscles during RSE (6 × 10‐s sprints with 30 s of passive recovery) with continuous BFR (40% AOP) compared to non‐BFR (Behrendt et al., 2023). However, analysis of EMG signal characteristics within a time domain (e.g., root mean square [RMS] or integrated EMG) is necessary to quantify muscle fatigue (Roman‐Liu et al., 2004). During a maximal voluntary contraction, EMG RMS of the elbow flexors did not differ following open‐looped arm‐cycling RSE (10‐s sprints with 20 s of active recovery) with continuous BFR (3‐cm wide cuffs inflated to 45% AOP) compared to non‐BFR (+18.5% vs. +18.2%, respectively) (Peyrard et al., 2019). These findings contrast with the traditional application of cuffs during low‐moderate intensity volume‐equated resistance exercise, where several studies have reported increased *biceps brachii* activation during elbow flexion tasks compared to non‐BFR (Wernbom et al., 2020). Thus, further investigation of muscle activation during volume‐matched RSE with BFR is warranted.

The current literature examines physiological responses during open‐looped (Peyrard et al., 2019; Willis et al., 2018; Willis, Borrani, & Millet, 2019; Willis, Peyrard, et al., 2019) or single‐set cycling RSE with BFR (Behrendt et al., 2023; Kojima et al., 2021; Wang et al., 2023), and/or often involves recreationally active participants (Behrendt et al., 2023; Ienaga et al., 2022; Peyrard et al., 2019; Wang et al., 2023; Willis et al., 2018; Willis, Borrani, & Millet, 2019; Willis, Peyrard, et al., 2019). However, trained team‐sport athletes typically perform RSE using a closed‐looped, multi‐set structure (Bishop et al., 2011), thus limiting the applicability of current findings. Therefore, the purpose of this study was to examine the impact of continuous BFR during practical RSE prescription on performance, peripheral, systemic physiological, and perceptual responses in team sport players. It was hypothesized that performance and muscle activation would be decreased, while muscle deoxygenation would be elevated during RSE with BFR compared to non‐BFR.

## 2 MATERIALS AND METHODS

Twenty‐six adult male team‐sport players volunteered for participation (age = 21 ± 5 years, height = 182 ± 8 cm, body mass = 81 ± 10 kg, and team sport experience = 15 ± 5 years), including 16 semi‐professional Australian Rules footballers from the same club and 10 amateur athletes (four Australian Rules footballers, four lacrosse players, one basketballer, and one touch rugby athlete). Male participants were exclusively chosen because they had the option to partake in a subsequent training study (data not presented herein; see Mckee et al., 2023) where menstrual cycle monitoring was not logistically feasible (Carmichael et al., 2021). Exclusion criteria included musculoskeletal injuries, hematological, or cardiovascular contraindications to BFR (Kacin et al., 2015). *A priori* power analysis using G*Power (version 3.1, RRID:SCR_013726, Heinrich‐Heine Universität Düsseldorf) determined that 26 participants would detect an effect size of 0.4 (Cohen's *d*_*z*_) between conditions and yield a power of 80% at an alpha level of *p* < 0.05 for mean power output during RSE (Beard et al., 2019). The study was approved by the Institutional Human Research Ethics Committee (2019/117) and performed according to the Declaration of Helsinki (2013), with participants signing written informed consent prior to testing.

A randomized crossover design was conducted, including one familiarization session, and two experimental sessions consisting of RSE with BFR or non‐BFR. Experimental sessions were separated by 4 ± 2 days and completed at the same time of day (within 2 ± 1 h). Participants were not permitted to perform high‐intensity exercise, or consume caffeine or alcohol, within 24 h prior to RSE sessions. The AOP assessment (158 ± 14 mmHg) and RSE familiarization were completed as previously described (Loenneke et al., 2012; Mckee et al., 2024), though participants warmed‐up at 1.0 W∙kg^−1^ of body mass on a mechanically‐braked cycle ergometer (Wattbike Ltd.) for five, rather than 10 min. Additionally, 10‐cm wide cuffs (SC10, Hokanson) applied bilaterally to the legs were inflated to 45% AOP (71 ± 6 mmHg) one second before sets using an E20 rapid cuff inflator and AG101 air source (Hokanson), and deflated immediately at the end of each set. Cuffs were inflated to 45% AOP as this was determined to be the highest tolerable pressure during pilot testing, which is in accordance with previous research (Giovanna et al., 2022; Mckee et al., 2024; Willis et al., 2018; Willis, Borrani, & Millet, 2019).

The two experimental sessions included warm‐up and gearing protocols identical to RSE familiarization. Sprints began from a stationary start at a 45° angle from the horizontal. The RSE included three sets of five 5‐s sprints with 25 s of passive recovery between repetitions and 3 min of rest between sets. These efforts were selected to replicate, but “overreach” typical sprints performed in team‐sport competition, which consist of a mean duration of 2–3 s (Spencer et al., 2005). Passive recovery periods of 25 s were utilized to ensure consistency with previous research incorporating closed‐looped cycling‐RSE with BFR using work:rest ratios between 1:4 to 1:6 (Ienaga et al., 2022; Kojima et al., 2021; Mckee et al., 2024; Wang et al., 2023).

Participants were instructed to remain seated during sprints to ensure performance repeatability for non‐cyclists (Reiser et al., 2002) and received strong verbal encouragement to give maximal effort. For the BFR session, cuffs were applied as described for familiarization though no cuffs were worn during the non‐BFR session. Power output was recorded continuously throughout the sessions at a frequency of 1 Hz using the Wattbike software (Expert v2.60.20). The sprint decrement (*S*_dec_) score for sets was calculated using the formula: % = (1 – [$\bar{S}$ × 5]/*S*_best_ × 5) × 100; where $\bar{S}$ is the mean power output of all sprints and *S*_best_ is the sprint with the highest mean power output (Girard et al., 2011). Average values for mean and peak power output were calculated for sets.

Muscle oxygenation of the left *vastus lateralis* muscle was monitored continuously using a PortaMon NIRS device (Artinis Medical System). The apparatus was secured to the muscle belly skin using double‐sided tape, positioned at two‐thirds of the distance from the anterior superior iliac spine to the patella. The device was wrapped in transparent plastic film to create a water‐proof barrier, and covered with black cloth to prevent contamination from exogeneous light sources. Transparent plastic sheets were used to mark the device location in reference to skin landmarks for consistent placement. Minimum TSI and concentration changes in oxyhemoglobin (ΔO_2_Hb), deoxyhaemoglobin (ΔHHb), and total hemoglobin (ΔtHb) were measured using continuous wavelengths of NIRS light (750 and 860 nm), and mean values during sprints and 25‐s rest periods were calculated for each repetition. All hemoglobin measures were normalized to express the magnitude of change from mean values obtained during a 3‐min baseline period (arbitrarily defined as 0 μM), where participants rested on the bike (Behrendt et al., 2023). Data included a standard differential pathlength factor of 4.0^7^ sampled at 10 Hz and was analyzed using the NIRS software (Oxysoft v3.0.53). A 0.5 s rolling average was used during analyses to smooth NIRS data (Rodriguez et al., 2018).

Surface EMG (Telemyo DTS, Noraxon) was used to assess activation of the right *vastus lateralis*, *biceps femoris*, and *lateral gastrocnemius* muscles. Participant's legs were shaved, abraded, and cleaned with alcohol at EMG sites to ensure low impedance. Bipolar silver/silver chloride surface electrodes (Myotronics, Inc.; diameter = 12.5 mm; inter‐distance electrode = 21 mm) were positioned over muscle bellies and transmitters were attached to the skin using double‐sided tape. Plastic transparent sheets were used for consistent EMG placement as described for NIRS. All EMG data were collected using the myoMUSCLE software (Noraxon) and sampled at 1500 Hz. A custom MATLAB script was used to process the data. Initially a fourth order zero‐lag band pass Butterworth filter was utilized with cut offs of 20 and 500 Hz. The EMG RMS was calculated over eight consecutive cycle revolutions for each sprint and normalized to values during the five‐second sprint from the warm‐up period (Girard et al., 2018).

Mean 15‐s V̇O_2_, minute ventilation (*V̇*_*E*_), and respiratory rate (RR) were measured continuously using a Parvo TrueOne 2400 metabolic cart (ParvoMedics) calibrated according to the manufacturer's recommendations. Arterial oxygen saturation (SpO_2_) and HR and were measured continuously at a frequency of 1 Hz using a pulse oximeter (Nonin WristOx_2_) and Garmin Edge 500 (Garmin), respectively. Fingertip capillary blood (0.3 µL) was assessed for blood lactate concentration (BLa^−^) via a handheld analyzer (Lactate Pro 2) after 2 min of passive rest following the final sprint in each set. Mean values for V̇O_2_, *V̇*_*E*_, RR, SpO_2_, and HR were calculated for sets, including both sprints and between‐sprint rest periods to account for delayed oxygen kinetics during intense cycling (Hill et al., 2003). In addition, average values for post‐set V̇O_2_ and *V̇*_*E*_ were calculated for 2 min following each set, while participants rested on the bike.

Modified CR‐10 Borg Scales were displayed during sessions to obtain rating of perceived exertion (RPE), limb discomfort, and breathing difficulty at the end of sets (same anchors ranging from 0 = “nothing at all” to 10 = “maximal”), and perceived recovery 15 s before sets (ranging from 0 = “very poorly recovered” to 10 = “very well recovered”) (Foster et al., 2001; Laurent et al., 2011). In addition, session RPE was collected 20 min after RSE completion (Foster et al., 2001). Before sessions, RPE, perceived limb discomfort, and breathing difficulty scales were explained to participants as “whole‐body exertion comprising any sensations in the legs or breathlessness during exercise”, “any uncomfortable exercise‐related sensations within the leg muscles”, and “the sense of breathlessness caused by exercise”, respectively (Peñailillo et al., 2018). Perceived recovery was defined as “the sense of overall physical and psychological recovery following exercise” (Laurent et al., 2011). Participants were instructed to report their perceptual responses relative to a self‐selected memory anchor, where the upper limit of scales represented the greatest level of exertion, limb discomfort, breathing difficulty, or recovery they have experienced during (or shortly following) any task (Malleron et al., 2023).

All data were determined to be normally distributed using Shapiro‐Wilk tests and are reported as mean ± standard deviation. Linear mixed models compared differences in dependent variable means between conditions (BFR and non‐BFR) and sets (one, two, and three) or repetitions (1–15). Conditions, sets, and repetitions were considered fixed factors, while participants were included as random factors. Main effects and interactions were examined using the Holm‐Bonferroni post hoc test. Condition, set, and repetition effect sizes were calculated using Cohen's *d*_*z*_ and classified using the scale developed by Hopkins et al. (2009) (0.20–0.59 = *small* effect, 0.60–1.19 = *moderate* effect, 1.20–1.99 = *large* effect, 2.0–3.9 = *very large* effect, and ≥4.0 = *extremely large* effect) (Cohen, 1988). Statistical analyses were conducted using SPSS (v24; IBM), with significance set to *p* < 0.05.

## 3 RESULTS

Key performance, physiological, and perceptual data during sets including interactions, main effects, and post hoc comparisons are shown in Table 1. For mean and peak power output and *S*_dec_ score, significant main effects were observed for condition and set (all *p* < 0.001), without interactions. Post hoc analyses indicated greater average mean and peak power output during non‐BFR compared to BFR (+5.3 ± 6.6%; *d*_*z*_ = 0.85 and +4.9 ± 6.5%; *d*_*z*_ = 0.77, respectively). Mean *S*_dec_ score was higher with BFR (8.9 ± 4.4%) than non‐BFR (6.8 ± 2.9%; *d*_*z*_ = 0.78).

**TABLE 1:** Mean (±SD) performance, physiological, and perceptual responses during sets one, two, and three of repeated‐sprint exercise with blood flow restriction (BFR) or without (non‐BFR).

	Set one	Set two	Set three	Condition	Set	Interaction
Performance						
Mean power output (W)^d^						
BFR^a^	895 ± 150	861 ± 135	835 ± 118	***p* < 0.001**	***p* < 0.001**	*p* = 0.738
Non‐BFR	933 ± 151	905 ± 148	886 ± 130			
Peak power output (W)^d^						
BFR^a^	990 ± 171	954 ± 156	932 ± 136	***p* < 0.001**	***p* < 0.001**	*p* = 0.686
Non‐BFR	1025 ± 172	1004 ± 166	984 ± 144			
*S*_dec_ score (%)^c^						
BFR^a^	7.0 ± 3.6	10.4 ± 4.7	9.4 ± 4.3	***p* < 0.001**	***p* < 0.001**	*p* = 0.253
Non‐BFR	5.7 ± 2.6	7.3 ± 2.7	7.4 ± 3.0			
Physiological						
V̇O_2_ (mL∙kg^−1^ min^−1^)^b^						
BFR^a^	26.4 ± 3.2	27.5 ± 3.6	26.4 ± 3.7	***p* < 0.001**	***p* = 0.026**	*p* = 0.286
Non‐BFR	28.1 ± 2.9	28.8 ± 3.8	28.8 ± 4.1			
*V̇*_*E*_ (L∙min^−1^)^c^						
BFR^a^	56.7 ± 13.0	65.3 ± 15.9	64.0 ± 14.2	***p* = 0.003**	***p* < 0.001**	*p* = 0.729
Non‐BFR	59.0 ± 12.7	66.3 ± 15.2	66.6 ± 15.1			
RR (breaths∙min^−1^)^d^						
BFR	31 ± 4	36 ± 6	37 ± 6	*p* = 0.510	***p* < 0.001**	*p* = 0.974
Non‐BFR	31 ± 4	35 ± 5	36 ± 6			
BLa^−^ (mmol∙L^−1^)^d^						
BFR	7.8 ± 2.5	9.3 ± 3.1	10.0 ± 3.4	*p* = 0.291	***p* < 0.001**	*p* = 0.905
Non‐BFR	7.8 ± 2.2	9.1 ± 3.0	9.7 ± 3.3			
HR (beats∙min^−1^)^c^						
BFR	142 ± 18	150 ± 18	151 ± 17	*p* = 0.057	***p* < 0.001**	*p* = 0.993
Non‐BFR	144 ± 16	151 ± 17	152 ± 16			
Perceptual						
Limb discomfort^d^						
BFR^a^	4.4 ± 1.7	5.6 ± 2.2	6.3 ± 2.7	***p* < 0.001**	***p* < 0.001**	*p* = 0.746
Non‐BFR	3.6 ± 2.0	4.6 ± 2.2	5.1 ± 2.6			
Breathing difficulty^d^						
BFR	4.2 ± 1.6	5.4 ± 2.0	5.8 ± 2.2	*p* = 0.304	***p* < 0.001**	*p* = 0.470
Non‐BFR	4.6 ± 1.9	5.4 ± 2.1	5.9 ± 2.4			
RPE^d^						
BFR	5.8 ± 1.6	6.6 ± 1.8	7.2 ± 1.9	*p* = 0.990	***p* < 0.001**	*p* = 0.635
Non‐BFR	5.6 ± 1.7	6.7 ± 1.7	7.4 ± 1.8			
Perceived recovery^d^						
BFR	9.1 ± 1.1	6.9 ± 1.7	6.0 ± 1.8	*p* = 0.499	***p* < 0.001**	*p* = 0.293
Non‐BFR	8.6 ± 1.5	6.9 ± 1.7	6.0 ± 1.8			

*Note*: Significant main effects are in bold.

Abbreviations: BLa^−^, blood lactate concentration; HR, heart rate; RPE, rating of perceived exertion; RR, respiratory rate; *S*_dec_, sprint decrement; *V̇*_*E*_, minute ventilation; V̇O_2_, oxygen consumption.

^a^ Significantly different to non‐BFR.

^b^ Set two is significantly different to set one.

^c^ Sets two and three are significantly different to set one.

^d^ All sets are significantly different.

**FIGURE 1:**
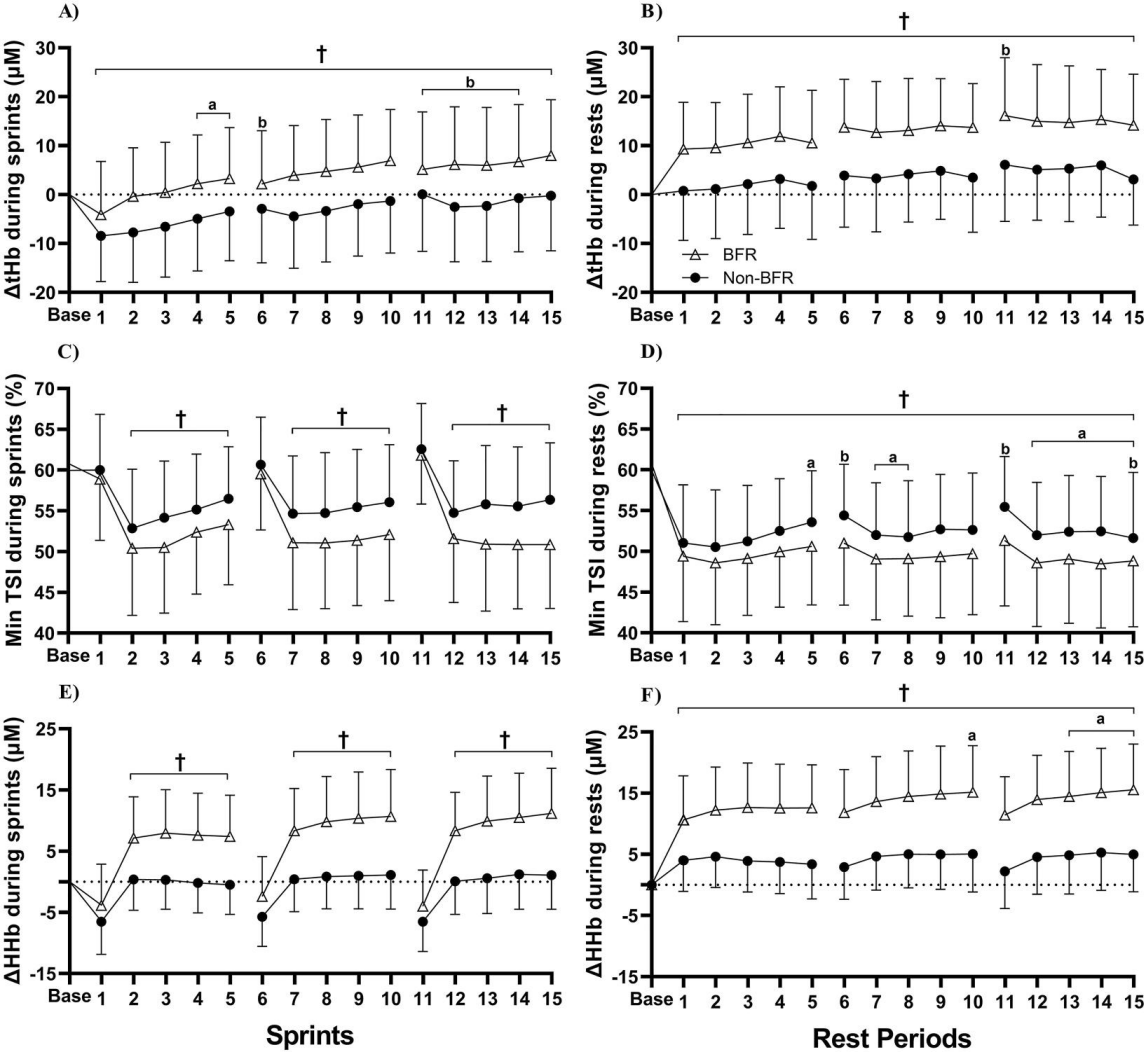
Concentration changes from resting baseline during sprints and rest periods for total hemoglobin (ΔtHb; panels (A and B), respectively), minimum tissue saturation index (min TSI; panels (C and D), respectively), and deoxyhaemoglobin (ΔHHb; panels (E and F), respectively) with blood flow restriction (BFR) or without (non‐BFR). †BFR is significantly different to non‐BFR (*p* < 0.05). ^a^Significantly different to the first repetition of the same set (*p* < 0.05). ^b^Significantly different to the same repetition in set one (*p* < 0.05).

Key NIRS data including interactions, main effects, and post hoc comparisons are presented in Figure 1. Regarding ΔtHb during sprints and rest periods, significant main effects were observed for condition and repetition (all *p* < 0.001), without interactions. Post hoc analyses indicated greater mean ΔtHb during sprints and rest periods for BFR (3.5 ± 10.9 and 12.9 ± 10.4 μM, respectively) compared to non‐BFR (−1.6 ± 9.0 μM; *d*_*z*_ = 0.58 and 4.3 ± 10.0 μM; *d*_*z*_ = 0.79, respectively). For minimum TSI and ΔHHb during sprints, significant condition × repetition interactions were observed (both *p* < 0.001). Post hoc analyses indicated lower mean minimum TSI and greater ΔHHb during the final four sprints of each set with BFR (51.4 ± 7.8% and 9.1 ± 7.1 μM, respectively) compared to non‐BFR (55.2 ± 6.9%; *d*_*z*_ = 1.26 and 0.5 ± 5.2 μM; *d*_*z*_ = 1.18, respectively). Regarding minimum TSI and ΔHHb during rest periods, significant main effects were observed for condition and repetition (all *p* < 0.001), without interactions. Post hoc analyses indicated lower mean minimum TSI and greater ΔHHb during rest periods for BFR (49.4 ± 7.1% and 13.7 ± 7.0 μM, respectively) compared with non‐BFR (52.5 ± 6.8%; *d*_*z*_ = 1.21 and 4.6 ± 5.7 μM; *d*_*z*_ = 1.41, respectively). For ΔO_2_Hb during sprints, significant main effects were observed for condition and repetition (both *p* < 0.001), without interaction. Post hoc analyses indicated greater mean ΔO_2_Hb during sprints for non‐BFR (−1.4 ± 7.0 μM) compared to BFR (−3.5 ± 10.4 μM; *d*_*z*_ = 0.27). Furthermore, mean ΔO_2_Hb was lower for the final four sprints compared to the first sprint of each set (*d*_*z*_ = 1.88). For ΔO_2_Hb during rest periods, a significant main effect was observed for repetition only (*p* < 0.001), with greater mean values during rest period 11 compared to rest periods 13 and 15 (*d*_*z*_ = 1.30 and 1.38, respectively).

**FIGURE 2:**
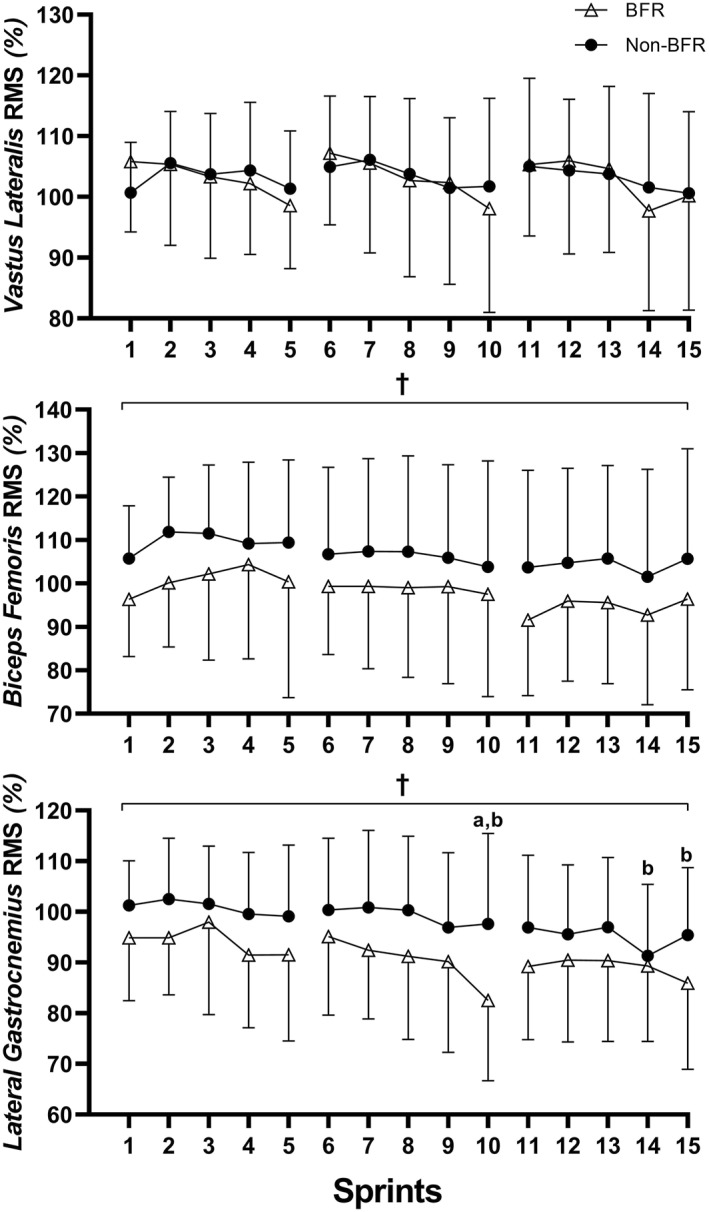
Electromyography root mean square (RMS) for (A) *vastus lateralis*, (B) *biceps femoris*, and (C) *lateral gastrocnemius* muscles during sprints with blood flow restriction (BFR) or without (non‐BFR). ^†^Non‐BFR is significantly greater than BFR (*p* < 0.05). ^a^Significantly lower than sprint 2 (*p* < 0.05). ^b^Significantly lower than sprint 3 (*p* < 0.05).

Data for EMG RMS including all main effects and post hoc comparisons are presented in Figure 2. For *vastus lateralis* EMG RMS, a significant main effect was observed for repetition only (*p* = 0.003), though post hoc analyses indicated no significant differences following Holm‐Bonferroni correction. Regarding *biceps femoris* EMG RMS, a significant main effect was observed for condition only (*p* = 0.007), with greater mean values during non‐BFR compared to BFR (+2.8 ± 18.9%; *d*_*z*_ = 0.35). For *lateral gastrocnemius* EMG RMS, significant main effects were observed for condition and repetition (both *p* < 0.001), without interaction. Post hoc analyses indicated greater *lateral gastrocnemius* mean EMG RMS during non‐BFR compared to BFR (+12.8 ± 13.6%; *d*_*z*_ = 0.79).

For V̇O_2_, a significant main effect was observed for condition and set (both *p* < 0.001), without interaction. Post hoc analyses indicated greater mean V̇O_2_ during non‐BFR compared to BFR (+6.9 ± 5.0%; *d*_*z*_ = 1.46). Regarding mean post‐set V̇O_2_, no differences (*p* = 0.803) were observed between BFR (16.5 ± 2.3 mL∙kg^−1^ min^−1^) and non‐BFR (16.5 ± 2.5 mL∙kg^−1^ min^−1^). For *V̇*_*E*_ and post‐set *V̇*_*E*_, significant main effects were observed for condition and set (all *p* < 0.02), without interaction. Post hoc analyses indicated greater mean *V̇*_*E*_ during non‐BFR compared to BFR (+3.7 ± 7.9%; *d*_*z*_ = 0.43). Furthermore, mean post‐set *V̇*_*E*_ was greater during BFR (47.4 ± 11.7 L∙min^−1^) compared to non‐BFR (46.3 ± 11.7 L∙min^−1^; *d*_*z*_ = 0.27), and during set two compared with set one (*d*_*z*_ = 0.97). For mean RR, BLa^−^, and HR, significant main effects were observed for set only (all *p* < 0.001). Regarding mean SpO_2_, no differences (*p* = 0.709) were observed between BFR (97 ± 1%) and non‐BFR (96 ± 2%).

For perceived limb discomfort, significant main effects were observed for condition and set (both *p* < 0.001), without interaction. Post hoc analyses indicated greater mean perceived limb discomfort during BFR compared to non‐BFR (*d*_*z*_ = 0.78). Regarding mean RPE, perceived breathing difficulty, and recovery, significant main effects were observed for set only (all *p* < 0.001). For mean session RPE, no differences (*p* = 0.846) were observed between BFR (6.6 ± 1.6) and non‐BFR (6.6 ± 1.5).

## 4 DISCUSSION

The purpose of this study was to examine the impact of continuous BFR during RSE on performance, peripheral, systemic physiological, and perceptual responses in team‐sport players. The main findings observed during RSE were: (1) reduced mean and peak power output, V̇O_2_, and *V̇*_*E*_ with BFR compared to non‐BFR, (2) further muscle deoxygenation and decreased *biceps femoris* and *lateral gastrocnemius* activation during BFR, though *vastus lateralis* EMG RMS did not differ between conditions, (3) comparable increases in BLa^−^, HR, and RPE between conditions, despite higher perceived limb discomfort during BFR. Altogether, these data suggest that RSE with BFR provides a more challenging physiological and perceptual stimulus for the periphery, at a reduced external power output, despite similar or reduced systemic demands.

Mean and peak power output were reduced (both −5%) and *S*_dec_ score was higher (+30%) with BFR compared to non‐BFR. These findings support emerging research indicating that BFR reduces external loads during RSE (Behrendt et al., 2023; Mckee et al., 2024; Willis et al., 2018). Performance decrements are expected with BFR from hypoxia‐mediated increases in muscle deoxygenation and metabolic stress, and reduced phosphocreatine supply (Behrendt et al., 2023; Harris et al., 1976). However, lower peak power output or increased fatigue indices are not consistently observed during RSE with intermittent or continuous BFR, despite some studies including greater sprint durations (6–10 s) and work:rest ratios (1:3–1:4) compared to the current research (Behrendt et al., 2023; Ienaga et al., 2022). These performance metrics could be maintained by manipulating other RSE variables to reduce fatigue accumulation (i.e., lowering total sprint volume [≤60 s] and/or increasing inter‐set rest period durations to 5 min) (Behrendt et al., 2023; Ienaga et al., 2022). Moreover, we previously reported that during RSE using an identical protocol, *S*_dec_ score and fatigue index were not different between BFR and non‐BFR for semi‐professional Australian Rules footballers (Mckee et al., 2024). These discrepant findings may be explained by the heterogenous sample in the current study, including amateur athletes who are likely less fatigue‐resistant and lacking experience with RSE and/or BFR compared to semi‐professional team‐sport players.

Decreased minimum TSI and greater ΔHHb and ΔtHb were observed during sprints and rest periods for BFR compared to non‐BFR. Additionally, ΔO_2_Hb (a proxy for oxygen delivery) was further reduced during sprints with BFR. However, ΔO_2_Hb is heavily influenced by rapid adjustments in blood volume during RSE (Rodriguez et al., 2018), which are exacerbated with cuff pressure. Therefore, considering that SpO_2_ did not differ between conditions, it can be suggested that BFR enhances localized but not systemic hypoxia during RSE. Greater ΔHHb has been reported as a proxy for enhanced oxygen extraction (Willis, Borrani, & Millet, 2019). However, caution is needed when interpreting this finding because corresponding increases in ΔtHb indicate that differences between conditions are more likely explained by greater vascular resistance and local blood pooling with BFR (Willis et al., 2018). In contrast, Kojima et al. (2021) observed comparable ΔHHb and ΔtHb during sprints and rest periods for RSE (5 × 10‐s sprints with 40 s of passive recovery) with intermittent BFR (140 mmHg during rest periods only) compared to non‐BFR. Therefore, continuous BFR appears superior to intermittent cuff application between sprints for exaggerating muscle deoxygenation which may provide a substantial stimulus for vascular adaptations to improve oxygen delivery or extraction.

Recruitment of additional fibers or increased firing frequency is commonly observed during sub‐maximal resistance exercise with BFR to compensate for fatigued fibers and sustain work (Wernbom et al., 2020). During RSE, *biceps femoris* and *lateral gastrocnemius* activation were reduced with BFR compared to non‐BFR, while *vastus lateralis* EMG RMS did not differ between conditions (Mendez‐Villanueva et al., 2012). This motor unit de‐recruitment may occur with fatigue if neural drive to active muscle is already maximized to meet RSE intensity demands. In contrast, *vastus lateralis* EMG RMS was increased during RSE (5 × 10‐s sprints with 60 s of passive recovery) with combined systemic hypoxia and BFR (fraction of inspired oxygen = 13.7%, and 140 mmHg inflation during rest periods, respectively) when compared to non‐BFR and the equivalent systemic hypoxia dose alone (Wang et al., 2023). However, EMG RMS data were normalized to the first sprint which was not completed in normoxia for all conditions (Wang et al., 2023), thereby limiting the reliability of comparisons between conditions. During a similar RSE protocol to ours (15 × 5‐s sprints with 25 s of passive recovery), *biceps femoris* and *lateral gastrocnemius* EMG RMS reduced in the 13th compared with first sprint, without changes for the *vastus lateralis* muscle (Hautier et al., 2000). Altogether, it appears that additional fatigue induced by BFR or condensing multi‐set RSE volume into a single‐set can decrease *biceps femoris* and *lateral gastrocnemius* activation. These findings suggest that BFR may modify motor unit recruitment strategies during cycling‐RSE, reducing coactivation of antagonistic muscles to optimize inter‐muscle coordination for power production given the probable deteriorated *vastus lateralis* contractility with metabolic stress (Hautier et al., 2000). However, EMG RMS of the *vastus lateralis* muscle decreased during a maximal voluntary contraction of the knee extensors following open‐looped RSE with BFR at 60% AOP (−36%) compared to non‐BFR (+9%) (Willis et al., 2018), which may be due to greater fatigue from higher cuff pressure or exercising until volitional exhaustion. Additional fatigue or compression with BFR could contribute to differences in muscle activation by altering cycling kinematics during RSE. Indeed, pedaling with the knee positioned further forward over the foot enhances quadriceps EMG RMS during moderate cycling intensities (180 W) (Tang et al., 2022), thus warranting further investigation during RSE with BFR.

Altered recruitment strategies during RSE with BFR may be impacted by differences in muscle fiber composition. In young men, superficial *vastus lateralis* muscles tend to have a higher percentage of type II fibers (∼68%) compared to *biceps femoris* (∼33%) and *lateral gastrocnemius* (∼57%) muscles (Johnson et al., 1973). Considering the maximal intensity demands and further muscle deoxygenation with BFR, there is likely a heightened reliance on type II fibers during RSE to compensate for the decreased contractility of oxygen‐deprived type I fibers (Mendez‐Villanueva et al., 2012). Unfortunately, this study did not include muscle biopsies, thus further research should determine if fiber type distribution impacts muscle activation during RSE with BFR (Tang et al., 2022). In our study, *vastus lateralis* and *biceps femoris* EMG RMS did not decrease in parallel with mechanical output across RSE, likely due to the mild (<10%) *S*_dec_ score observed for both conditions (Girard et al., 2011). Therefore, our findings support previous research dissociating muscle activation from subsequent sprint performance during RSE, indicating that metabolic stress likely better explains reduced mechanical output (Mendez‐Villanueva et al., 2012).

We observed comparable increases in BLa^−^ between conditions, which is common during RSE, regardless of whether cuffs remain inflated during assessment (Behrendt et al., 2023; Ienaga et al., 2022; Mckee et al., 2024). This consistent BLa^−^ increase between conditions could be interpreted as similar anaerobic energy contribution during RSE (Sahlin, 2014). However, it is worth noting that the same BLa^−^ was achieved with BFR despite lower mechanical output compared to non‐BFR. Moreover, post‐set *V̇*_*E*_ was increased for BFR, likely to expel excess carbon dioxide produced from greater anaerobic glycolysis contribution (Mckee et al., 2024). Considering that post‐set V̇O_2_ did not differ between conditions, a greater relative proportion of V̇O_2_ and blood flow was possibly redistributed to respiratory muscles when using inflated cuffs to meet metabolic demands (Harms et al., 1997). In contrast, V̇O_2_ and *V̇*_*E*_ during sets were lower for BFR, which is likely due to the lower mechanical output compared to non‐BFR. Despite the lower external load with BFR, HR was comparable between conditions. This could be explained by local blood pooling with BFR or hypoxia‐induced stimulation of the muscle metaboreflex, leading to greater sympathetic nerve activity and HR for a given power output (Conceição et al., 2018). Indeed, when mean power output is comparable during RSE, BFR increases HR compared to non‐BFR (Kojima et al., 2021). Therefore, BFR likely increases the anaerobic demands of RSE, and may enhance cardiac output for a given power to compensate for reduced oxygen availability at the muscle.

Perceived limb discomfort was greater with BFR compared to non‐BFR, while RPE and perceived breathing difficulty increased similarly between conditions, likely due to the maximal intensity demands of RSE. In support of our findings, perceived limb discomfort was higher and RPE was not significantly different for elite badminton players completing running‐RSE (3 sets of 10 × 10‐s sprints with 20 s of passive recovery and 3 min of rest) with continuous BFR (40% AOP) compared to non‐BFR (Valenzuela et al., 2019). Greater perceived limb discomfort is commonly observed during RSE with BFR and can be attributed to cuff compression and metabolite accumulation (Willis et al., 2018; Willis, Borrani, & Millet, 2019). Despite the greater perceptual stimulus at the periphery, perceived recovery and session RPE did not differ between conditions, indicating that RSE with BFR is well tolerated by team‐sport players.

This study is not without limitations. Firstly, the accuracy of NIRS measures can be influenced by differences in adipose tissue thickness and variations in skin perfusion during exercise (Rodriguez et al., 2018). Furthermore, surface EMG can be impacted by motor unit cycling during fatiguing contractions, which may be enhanced as BFR increases the reliance on highly‐fatigable type II fibers, thus, muscle activation cannot be directly inferred by EMG RMS (Girard et al., 2011). Finally, greater sprint durations and work:rest ratios (10‐s efforts with a 1:2 work:rest ratio) during RSE with systemic hypoxia reduced performance and *vastus lateralis* muscle oxygenation compared to a similar protocol to ours (5‐s efforts with a 1:4 work:rest ratio) (Dennis et al., 2023). It is possible that higher work:rest ratios of 1:2 during RSE provide a preferred “trade‐off” between internal and external loads to stimulate physiological adaptations for team‐sport players. Therefore, further research should examine the impact of work:rest ratios during RSE with BFR on performance, physiological, and perceptual responses.

## 5 CONCLUSION

In conclusion, RSE with BFR increased the physiological stress (further muscle deoxygenation and reduced muscle activation) and perceived limb discomfort at the periphery, which likely reduces performance by enhancing anerobic energy production. However, BFR induced comparable (BLa^−^, HR, and RPE) or reduced (V̇O_2_ and *V̇*_*E*_) systemic demands and is tolerated similarly to non‐BFR by team‐sport players. Further research should refine the structure of multi‐set closed‐looped RSE with BFR to optimize the physiological stress for promoting adaptations in team‐sport players.

## CONFLICT OF INTEREST STATEMENT

The authors declare no conflicts of interest.

## Data Availability

Research data are not shared.
